# Manufacturing processes, additional nutritional value and versatile food applications of fresh microalgae *Spirulina*

**DOI:** 10.3389/fnut.2024.1455553

**Published:** 2024-09-04

**Authors:** Guanghong Luo, Haiyan Liu, Shenghui Yang, Zhongliang Sun, Liqin Sun, Lijuan Wang

**Affiliations:** ^1^Gansu Engineering Technology Research Center for Microalgae, Hexi University, Zhangye, China; ^2^College of Life Sciences, Yantai University, Yantai, China

**Keywords:** fresh spirulina, photobioreactor, nutritional merits, food processing, phycocyanin

## Abstract

*Spirulina* is capable of using light energy and fixing carbon dioxide to synthesize a spectrum of organic substances, including proteins, polysaccharides, and unsaturated fatty acids, making it one of the most coveted food resources for humanity. Conventionally, *Spirulina* products are formulated into algal powder tablets or capsules. However, the processing and preparation of these products, involving screw pump feeding, extrusion, high-speed automation, and high-temperature dewatering, often result in the rupture of cell filaments, cell fragmentation, and the unfortunate loss of vital nutrients. In contrast, fresh *Spirulina*, cultivated within a closed photobioreactor and transformed into an edible delight through harvesting, washing, filtering, and sterilizing, presents a refreshing taste and odor. It is gradually earning acceptance as a novel health food among the general public. This review delves into the manufacturing processes of fresh *Spirulina*, analyzes its nutritional advantages over conventional algal powder, and ultimately prospects the avenues for fresh *Spirulina’s* application in modern food processing. The aim is to provide valuable references for the research and development of new microalgal products and to propel the food applications of microalgae forward.

## 1 Introduction

*Spirulina*, also known as *Arthrospira*, is a type of blue-green algae that has been consumed by humans for centuries. Historical records reveal its use as a food source by the Aztecs and other Mesoamerican cultures dating back to the 16th century. Traditionally, it was harvested from the alkaline waters of Lake Texcoco in Mexico and transformed into dried cakes for consumption (1, 2). Today, *Spirulina* stands as a prominent economic microalgae species, produced extensively worldwide, and has become a favored dietary supplement among health-conscious individuals. This remarkable algae species is abundant in high-quality plant proteins, polysaccharides, carotenoids, phycocyanin, vitamins, minerals, and other trace elements (3). Research has uncovered a multitude of health benefits associated with *Spirulina*, including its ability to lower blood lipids, resist oxidation, combat infection, cancer, radiation, and aging, while also enhancing the body’s immune system. Consequently, *Spirulina* presents itself as an exceptional candidate for the creation of high-quality dietary supplements and nutraceutical products (4, 5).

Currently, *Spirulina* is commercially produced in diverse locations worldwide, including Hawaii, Mexico, Thailand, China, and India. The cultivation process entails growing the algae in shallow water and constantly agitating the water to ensure optimal sunlight exposure and nutrient absorption (4). Once harvested, *Spirulina* can be transformed into various forms such as dry powder, flakes, tablets, and capsules, making it a versatile dietary supplement. However, in the conventional production process, after harvesting and spray drying, the fresh biomass undergoes high temperatures, leading to significant nutritional losses. This includes protein denaturation, pigment oxidation, and Maillard reactions between sugar and amine, ultimately resulting in reduced nutritional value. Specifically, carotenoids and phycocyanin levels decrease by over 20%, and other heat-sensitive biological enzyme active substances also experience substantial losses (6, 7).

Therefore, the living and fresh *Spirulina* presents itself as an exceptional alternative to the currently available dry powder products in the market, particularly appealing to consumers who are increasingly mindful of their nutrition. The fresh *Spirulina* is primarily cultivated in closed photobioreactor, eliminating the need for spray drying and thus fully preserving its nutrient composition and bioactive substances. Its refreshing and odorless taste further enhances its appeal to the public (8). China has emerged as a leader in the design and development of fresh *Spirulina*, solidifying its position as the world’s largest producer. Concurrently, other major algae-producing countries have also demonstrated remarkable efforts and achievements, having developed a diverse range of fresh *Spirulina* products (9, 10). However, a comprehensive understanding of the nutritional value and application potential of fresh *Spirulina* remains untapped. This review aims to compare fresh *Spirulina* with traditional *Spirulina* products, analyze its nutritional advantages, and summarize its application and development prospects in food processing. By doing so, it lays a foundation for the research of related products and the advancement of the microalgal industry.

## 2 The production processes differences between fresh *Spirulina* and traditional product

**Figure 1:**
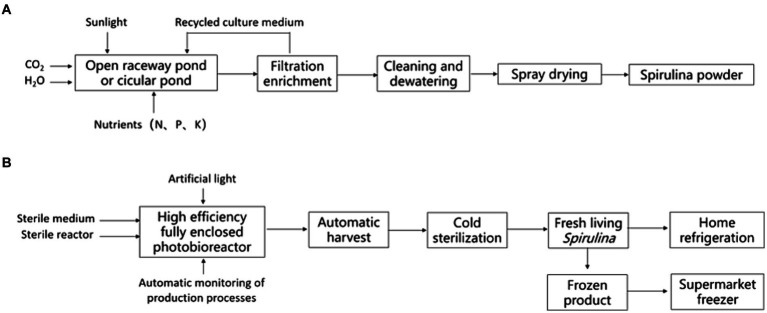
Process flow chart of *Spirulina* powder **(A)** and fresh *Spirulina* **(B)**.

The two forms of *Spirulina* products, fresh *Spirulina* and *Spirulina* powder (or tablets and pressed candies made from processed *Spirulina* powder), exhibit notable differences in production process, preservation, and nutritional composition. Traditional *Spirulina* products are derived from artificially large-scale cultivated *Spirulina platensis* or *Spirulina maxima*, undergoing harvest, leaching, rinsing, and drying (1). Fresh *Spirulina*, on the other hand, refers to concentrated slurry obtained by simply cleaning and processing fresh *Spirulina* cells harvested from closed photobioreactors. It can be consumed directly or added to food (8, 11) (Figure 1).

### 2.1 Cultivation systems

**Figure 2:**
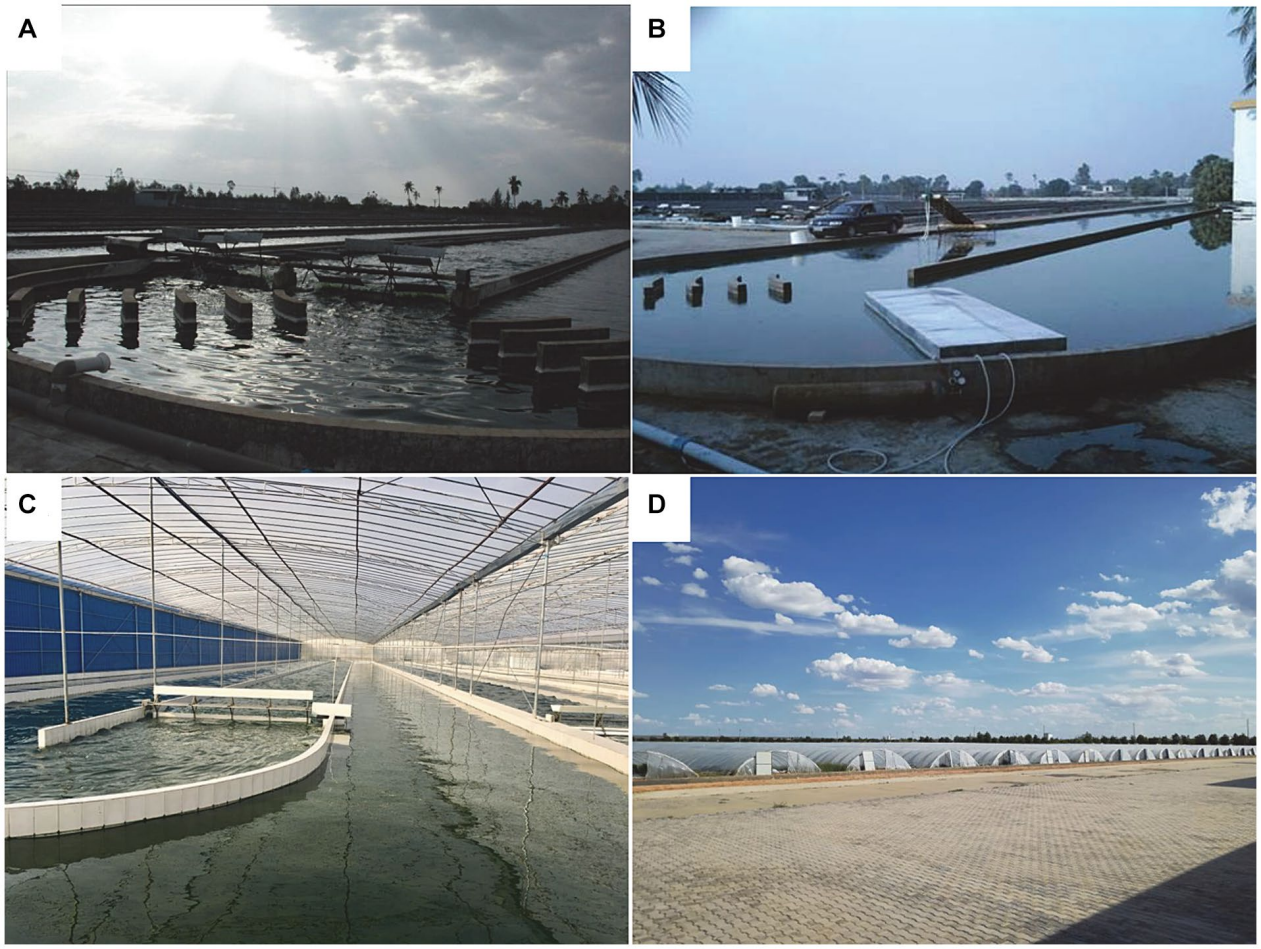
Microalgae large-scale cultivation. **(A)** Raceway pond for *Spirulina* production in Sanya. **(B)** Anti-leakage equipment for CO_2_ supplementation. Raceway pond in greenhouse in Inner Mongolia greenhouse interior **(C)** and exterior **(D)**.

Currently, the dry powder is mostly produced using open raceway pond systems, such as those employed by Earthrise Nutritionals, Cyanotech Corporation in the United States, and Neptunus Corporation in China (Figures 2A,B). The Chinese scientists and entrepreneurs have innovatively invented greenhouse raceway pond technology, which represents the most critical technological breakthrough in the industrialization of *Spirulina* production (Figures 2C,D). This technology significantly extends the annual production period and has directly led to a leap in the biomass of *Spirulina* in China (12). These cultivation systems utilize natural lighting and employs paddle wheel mechanisms to drive liquid flow and mixing, ensuring uniform light exposure for the *Spirulina*. Due to its fully or partially open environment, the production process of *Spirulina* is susceptible to external environmental influences such as rainwater, dust, bacteria, and zooplankton. These contaminants may potentially impact the quality and safety of *Spirulina*.

The production of fresh *Spirulina* usually adopts full-enclosed photobioreactors that are ideal for controlling the growth environment. In a fully enclosed reactor, light intensity, temperature, nutrient supply, and gas composition can be adjusted to maximize the productivity and quality of the microalgal biomass. Moreover, strict aseptic operations and equipment disinfection measures inside the reactor are required to ensure sterility. The filtration of air is also crucial for aseptic operation, generally achieved through high-efficiency filters and air disinfection equipment (10, 13).

**Figure 3:**
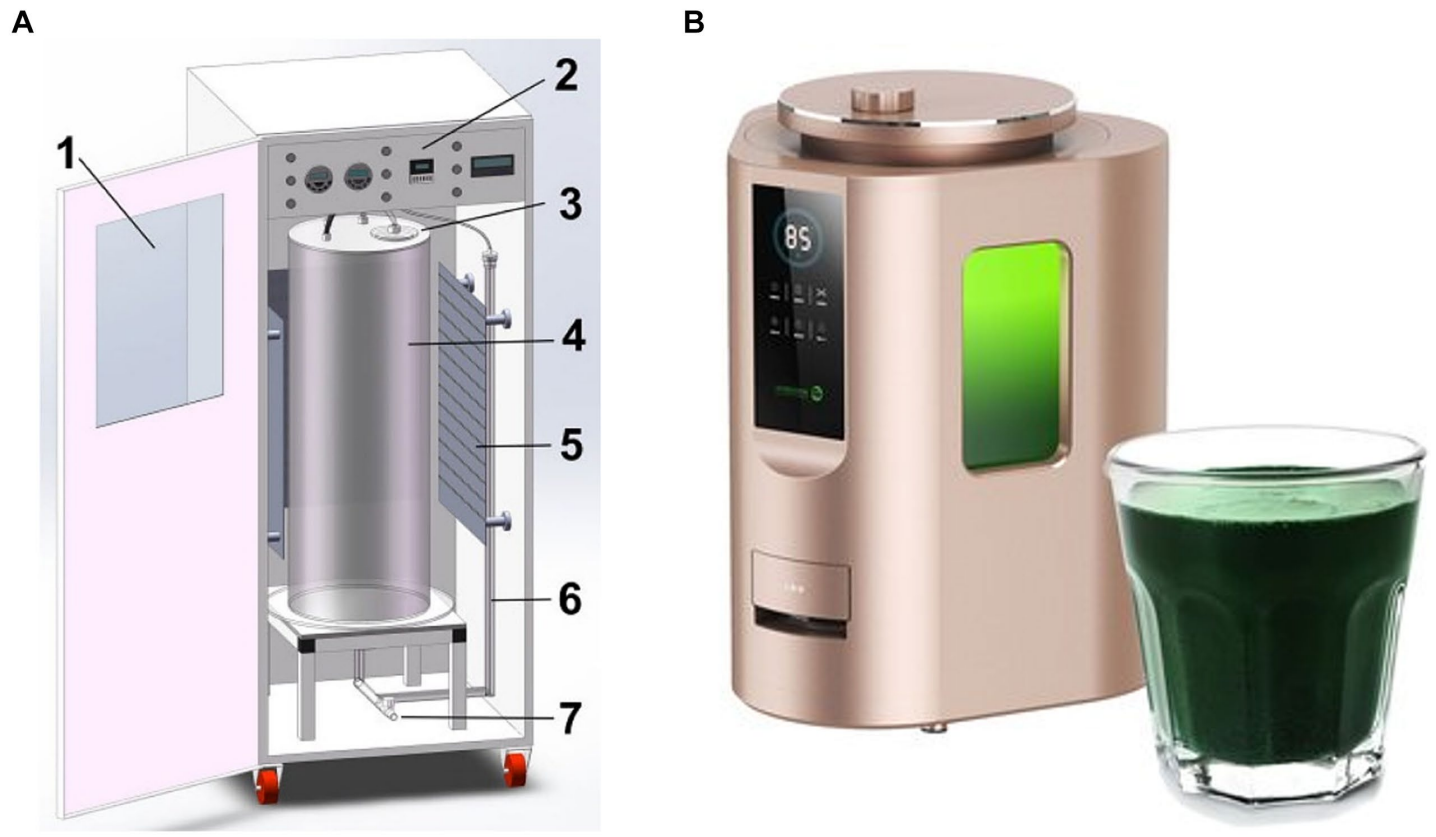
Schematic diagram of column bioreactor for fresh *Spirulina* production (**A**, 1: observatory window and front door; 2: controlling panel; 3: reactor upper cover; 4: reactor column; 5: LED light source panel; 6: aeration pipe; 7: water tap) and commercially available equipment **(B)**.

Photobioreactors are usually composed of vertically or horizontally combined pipes (made of borosilicate or organic glass). In the reactor system with horizontal pipelines, the circulation of the culture medium is driven by pumps. The mixed gas with a high content of CO_2_ is introduced intermittently or in the pH-feedback-style in the gas–liquid exchange unit to supplement the carbon source for *Spirulina* growth. Reactors with vertical pipes have a relatively large diameter, usually between 30 and 60 cm. Some light sources of reactors are located inside the cylinder, and others are placed around the reactor to enhance the photosynthesis of microalgal cells. This type of reactor has a high ventilation intensity, requiring extremely rigorous air sterility (Figure 3).

Fresh *Spirulina* and its production facilities have potentially vital applications in life support systems, especially in enclosed environments, such as space stations and submarines (14, 15). During growing, *Spirulina* utilizes light energy to absorb CO_2_ and release oxygen, which is instrumental in maintaining good air quality in enclosed environments such as space stations. Furthermore, *Spirulina* can provide abundant nutrients, including proteins, vitamins, and minerals, preventing individuals in enclosed environments from nutritional deficiencies, especially vitamin deficiencies. In summary, fresh *Spirulina* can serve as a nutritious food source, contributing to resource cycling and the self-sustaining of ecosystems in long-term hermetic missions.

### 2.2 Hygiene and quality problems

Large-scale cultivation of *Spirulina* typically involves outdoor open ponds driven by paddle wheel mechanisms. These systems are susceptible to contamination by pathogenic microorganisms, including bacteria, fungi, viruses and predators, such as protozoa, rotifers, copepods and cladocerans. Indeed, contamination poses a significant challenge, as it has the potential to substantially diminish the quality of *Spirulina* products and may potentially lead to foodborne illnesses. In addition, the contamination also reduces the growth rate and the overall biomass productivity. Thus, effective biosecurity and quality control measures during the production process are crucial for preventing biological contamination and ensuring product safety and quality (9).

The methods to control contamination during the cultivation of *Spirulina* mainly include chemical and physical methods, such as adding antibiotics, ammonium salts (16), as well as filtration or foam flotation (17). To guarantee safety and long-term shelf-life of the dietary supplement, the fresh biomass harvested from open pond is usually hot or spray dried at temperatures above 100°C. Nevertheless, due to contamination during the cultivation process, some bacterial or fungal toxins may remain in the dried *Spirulina* powder, or the ash content of the *Spirulina* dry powder may increase. According to the Chinese National Standard (GB/T 16919–2022 General Quality Rules for Edible Spirulina Powder), edible *Spirulina* dry powder must not be detected with mycotoxins, and its ash content should be less than 7%.

The hygienic quality of fresh *Spirulina* primarily relies on the sterilization efficacy of the culture medium and equipment, pollution control techniques during cultivation, and non-thermal sterilization methods after filtration and concentration (9). Fresh *Spirulina* is typically cultivated using purified mineral water or pure water supplemented with nutrient salts as the culture medium. The cultivation equipment is primarily made of high-borosilicate glass or stainless steel that can withstand high-temperature sterilization. Due to the adoption of a fully closed cultivation system, clean air filtration ensures minimal microbial growth. According to the “Fresh *Spirulina*” group standard (QMZWZ [2022] No. 5) issued by the Qingdao Microalgae Industry Association, the total bacterial count in fresh *Spirulina* for direct consumption should be less than 10,000 CFU/g, and harmful bacteria such as *Salmonella* and *Staphylococcus aureus* should not be detected. When stored in a light-blocking, well-packaged condition at −18°C (±2°C), fresh *Spirulina* has a shelf life of 12 months, while homemade products should be consumed promptly.

### 2.3 Downstream processing

**Figure 4:**
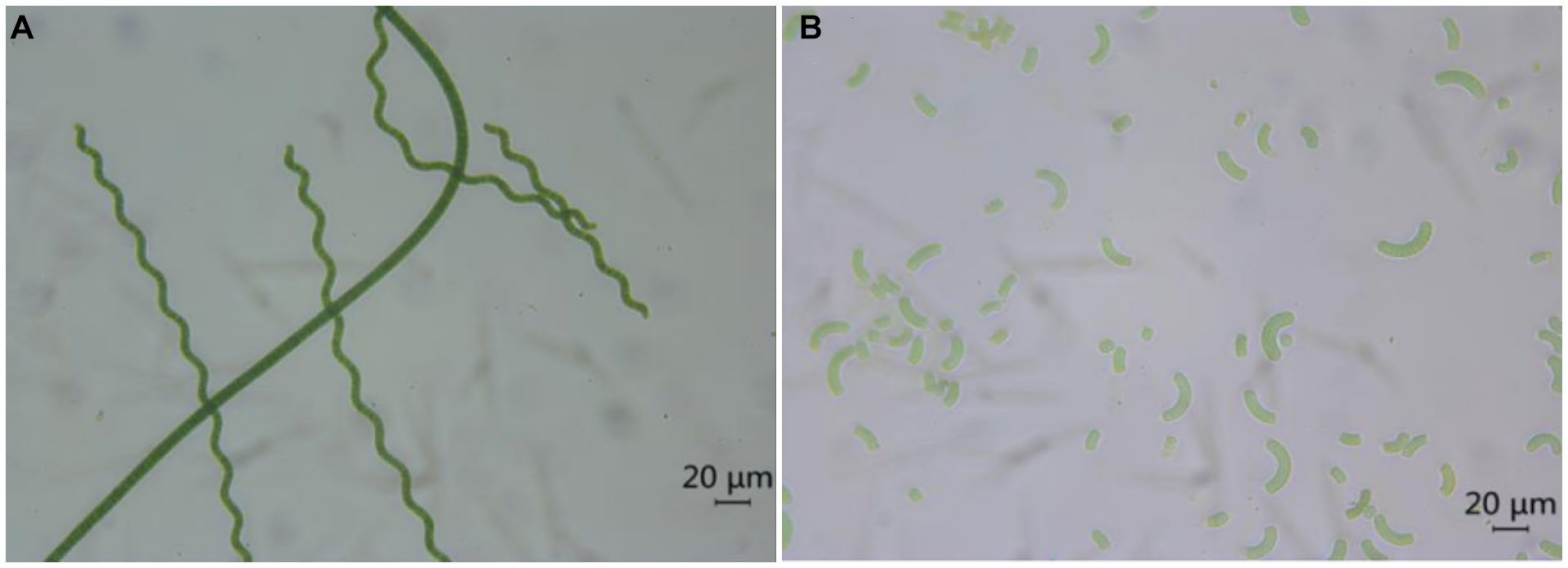
Optical photos of fresh *Spirulina* **(A)** and redissolved *Spirulina* powder **(B)** under a microscope.

*Spirulina* is typically dried after harvesting to facilitate its storage and incorporation into food. The drying of *Spirulina* accounts for approximately 30% of the total production cost, and the traditional methods employed to dry fresh *Spirulina* include spray drying, freeze drying, solar drying, convective hot air drying, and spouted bed drying (18). Subsequently, after re-dissolution, the algal powder presents evident breakage and damage under the microscope. In contrast, the algal filaments of fresh *Spirulina* are intact, without obvious breakage, damage, or leakage of ingredients (Figure 4).

Currently, the primary decontamination methods for fresh *Spirulina* involve non-thermal sterilization technologies such as pulsed electric field (PEF), ultrasonication, ozonation and cold plasma (19, 20). Within this array of approaches, each method exhibits unique strengths and limitations (as outlined in Table 1), thereby contributing to the ongoing quest for an optimal decontamination solution for *Spirulina* biomass (9). While preserving nutritional and sensory attributes, these techniques avoid the drawbacks of traditional thermal methods. Cold plasma, in particular, stands out for its versatility, efficacy, and ability to maintain *Spirulina’s* nutritional excellence. However, challenges persist, including equipment costs and the need for further research to optimize its application at large scales. By carefully selecting the appropriate method, the industry can produce safer and higher-quality fresh *Spirulina* products.

**Table 1:** Comparison of major advantages and disadvantages of non-thermal techniques for fresh *Spirulina.*

Methods	Advantages	Disadvantages
Pulsed electric field	Apply liquid and some solid foods. Preserves nutrition and sensory attributes.	Limited effectiveness to certain microbes. High energy requirement and cost. Toxicity due to electrode corrosion.
Ultrasonication	Apply to solid and liquid foods. Improved product quality attributes.	Effectiveness varies against spores. May cause cavitation-induced damage in nutrient structure.
Ozonation	Inexpensive	Careful handling required due to highly reactive nature. Effectiveness varies. Formation of potential harmful by-product during process.
Cold plasma	Apply to solid and liquid foods. Reaches areas difficult to access. Preserve nutrition and sensory attributes of foods.	Technology is still immature. Requires equipment. Cause changes in nutrients.

### 2.4 Nutrient loss caused by conventional drying method

The dehydration and drying processes of *Spirulina* powder may induce the loss of some nutritional components. In particular, some thermal-sensitive nutrients, such as vitamins and enzymes, may degrade or denature during processing. By comparison, without intense processing, more natural nutrients are retained in fresh *Spirulina*. The nutritional components affected by processing means include but are not limited to the following four categories.

#### 2.4.1 Loss of heat-sensitive nutrients

*Spirulina* contains various heat-sensitive nutrients, such as vitamins C and B vitamins. Because of the high temperature during drying (the spray drying temperature is usually 180–200°C for inlet air and 80–90°C for outlet air), these nutrients are partially decomposed or volatilized, declining their content in *Spirulina* powder (18). Regarding B vitamins, the total content of B1, B2, niacin, and B6 in fresh *Spirulina* is 275 mg/kg (on a dry basis), while that in *Spirulina* powder is 216 mg/kg (on a dry basis), lost by about 20% (21).

#### 2.4.2 Abated protein and enzyme activity

*Spirulina* is rich in proteins and enzyme substances, some of which are critical for human health. However, the high temperature in the drying process may cause the denaturation of some proteins and loss of enzyme activity, affecting the content and activity of these nutrients in *Spirulina* powder. The activity of superoxide dismutase is not detected in *Spirulina* powder, while its activity unit is 167.7 U/g in fresh *Spirulina*. In an experiment of feeding mice with fresh *Spirulina* and dried algae powder, the serum content of SOD with anti-lipid peroxidation function in the fresh *Spirulina* group is significantly higher than in the algae powder group, indicating that fresh *Spirulina* has a superior effect on the body’s antioxidant function (22).

#### 2.4.3 Oxidation and degradation of lipids

*Spirulina* consists of abundant unsaturated fatty acids and other lipid substances, possessing substantial nutritional value and health functions (23). However, the drying process brings about the oxidation and degradation of some lipids, diminishing the content of unsaturated fatty acids in *Spirulina* powder. For instance, eicosapentaenoic acid plays a positive role in lowering blood lipids; docosahexaenoic acid is a major nutrient for the growth and maintenance of nervous system cells and an essential fatty acid that makes up the brain and retina, commonly known as “brain gold.” They are barely detected in *Spirulina* powder, while in fresh *Spirulina*, their concentrations reach 4.2 mg/100 g (algal slurry) and 1.9 mg/100 g (algal slurry), respectively (24).

#### 2.4.4 Loss of active ingredients

Some active ingredients in *Spirulina*, such as polysaccharides and antioxidants, may be impacted by drying treatment. High temperatures and the drying process can cause the denaturation or deactivation of some active ingredients, thereby affecting their content and biological activity in *Spirulina* powder. Phycocyanin is a natural blue pigment with an outstanding antioxidant capacity and an active protein abundant in *Spirulina*. After drying at a high temperature of 200°C, the total content of phycocyanin and allophycocyanin in *Spirulina* powder drops by nearly 20% (6, 25).

## 3 Nutritional advantages and health benefits of fresh *Spirulina*

### 3.1 Amino acid composition and nutritional evaluation of fresh *Spirulina*

Fresh *Spirulina* boasts a protein content of no less than 5.5 g per 100 g, surpassing human milk by 6-fold, cow’s milk by 2-fold, and even outpacing goat’s milk by 14%. Moreover, it is abundant in various nutrients essential for a balanced, modern diet, as outlined in Table 2 (2, 9).

**Table 2:** Comparison of animal milk and fresh *Spirulina* (g/100 g).

Component	Human milk	Cow milk	Goat milk	Fresh *Spirulina*
Water	80	80	81.6	89
Crude protein	1.12	3.2	5.7	6.5
Carbohydrates	7.4	3.8	5	1.65
Lipid	3.4	3.9	7.1	0.6
Crude fiber	0	0	0	0.05

The protein content of fresh *Spirulina* encompasses a broad spectrum of essential amino acids, making it a valuable source of plant-based protein (26). Its supply of essential amino acids is nearly identical to the recommended nutritional composition pattern for humans by the Food and Agriculture Organization of the United Nations and the World Health Organization. Compared with natural cow’s milk, fresh *Spirulina* may exhibit varying levels and ratios of specific essential amino acids. For example, it is abundant in lysine and isoleucine and relatively deficient in histidine and tryptophan. However, fresh *Spirulina* stands out as a nutrient-rich, low-fat, and low-cholesterol food alternative to cow’s milk. Moreover, it serves as a superior protein source for vegetarians and lactose-intolerant individuals due to its enhanced digestibility and absorption rates (27).

Overall, the amino acid composition and supply of fresh *Spirulina* make it a meritorious source of protein, especially for vegetarians and those seeking various protein intakes. Its amino acid composition is more comprehensive than that of natural milk, enhancing its nutritional value.

### 3.2 Content and unique health functions of phycocyanin

Phycocyanin is one of the most active ingredients in fresh *Spirulina* and has a strong antioxidant ability. Its content in *Spirulina* varies with the variety, growth conditions, and harvest timing, typically accounting for 2–10% of total proteins in *Spirulina* (28). However, the drying process can result in a loss of phycocyanin. Particularly, high temperatures and prolonged intense light may lead to partial denaturation or degradation of phycocyanin (7, 25). In contrast, the complete cell structure in fresh *Spirulina* can effectively diminish the wastage of phycocyanin as a natural barrier. The efficacy and application areas of phycocyanin include but are not limited to the following types.

#### 3.2.1 Antioxidant

Phycocyanin helps neutralize free radicals in the body and reduce cellular oxidative stress to prevent multiple chronic diseases. When alleviating the side effects of the anticancer drug doxorubicin, phycocyanin can downgrade the expression of the apoptotic gene Bax and the release of cytochrome C in myocardial cells, thereby lowering the oxidative damage of doxorubicin to myocardial cells and abating the risk of triggering primary cardiomyopathy and congestive heart disease (29). Moreover, phycocyanin can serve as an antioxidant in food, health products, and cosmetics.

#### 3.2.2 Cancer treatment

Phycocyanin has shown certain potential in cancer treatment. Some studies suggested that it might constrain tumor growth and spread and alleviate the side effects of radiotherapy and chemotherapy. Phycocyanin can inhibit the proliferation of breast cancer cell MCF-7 *in vivo* and *in vitro* and promote cell differentiation and apoptosis (30). Additionally, phycocyanin can suppress the proliferation of ovarian cancer cell SKOV-3 by mediating vascular endothelial growth factor (VEGF) and the signaling pathway of oncogene p53 (31). It can enhance the efficiency of cancer cell therapy by reducing the expression of multidrug-resistant gene proteins. The above findings provide important research directions for the treatment of multiple malignant tumors, new ideas for exploring targeted, safe, and efficient tumor treatments, and broad market prospects for the development and application of fresh *Spirulina*.

#### 3.2.3 Immune regulation

Phycocyanin is believed to have a specific immune regulatory effect, which can improve the body’s immune function, strengthen resistance, and abate the risk of potential autoimmune diseases. Liu et al. revealed that after treating allergic mice with phycocyanin, the levels of IgE and histamine in the mice decreased, and the release of cytokines IL-4 and IL-13 was downregulated, verifying that phycocyanin can reduce allergic reactions in mice through its immunomodulatory activity (32). Chang et al. confirmed that phycocyanin could decline cellular endocytosis and elevate the expression of costimulatory molecules (33).

#### 3.2.4 Anti-inflammation

**Figure 5:**
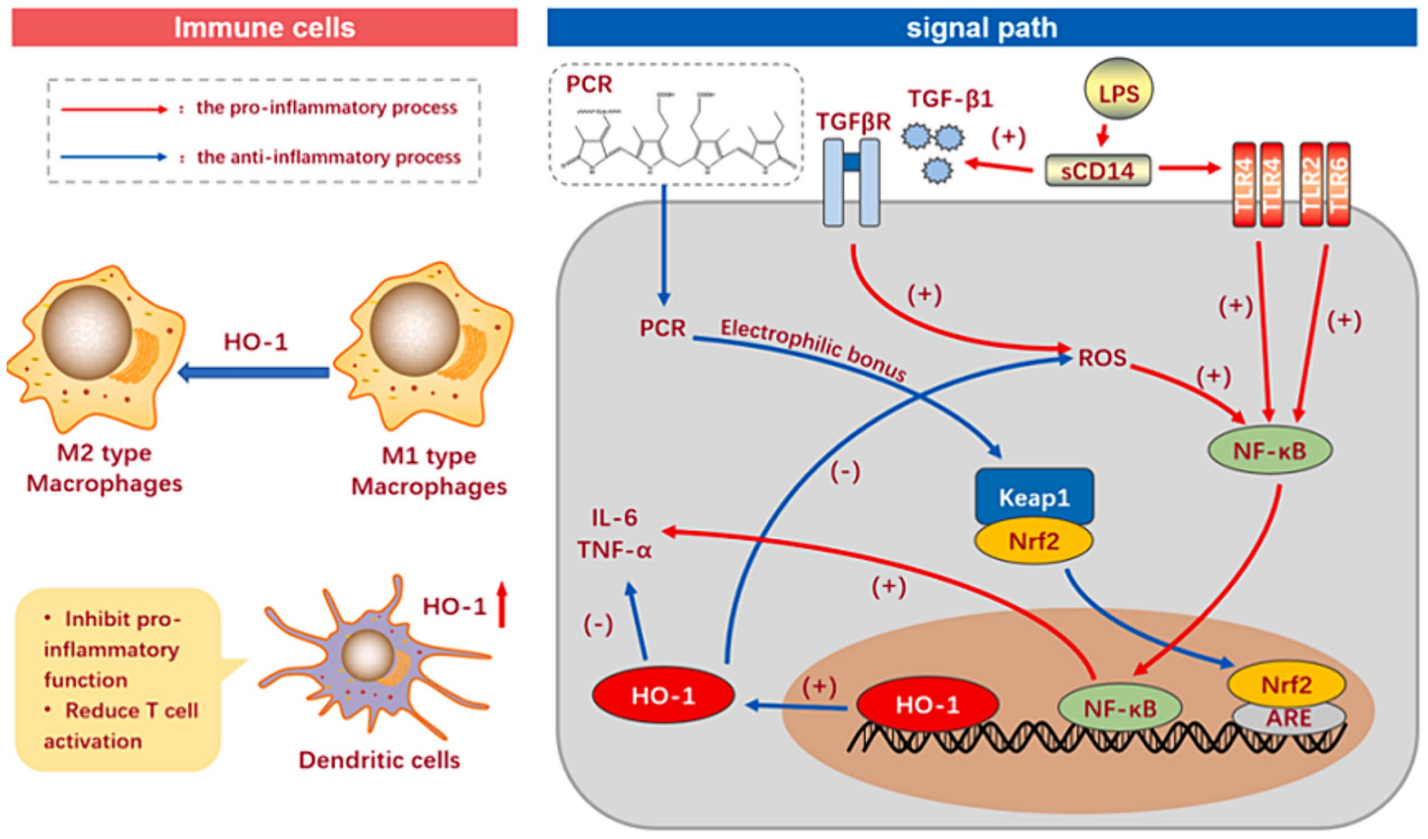
The anti-inflammatory mechanism of phycocyanin.

Inflammation is a dynamic feed-back of the autoimmune system, which can promote the recovery of damaged tissues, eliminate aging and diseased cells, and protect oneself from external invasion of viruses and bacteria. However, excessive inflammation attacks the tissues and cells of important organs while clearing pathogens, leading to pathological changes and disrupting health (34, 35). Phycocyanin can directly restrain inflammation. The action modes include TLR and KF- κB pathways. Meanwhile, it can indirectly curb inflammation by acting on signaling pathways such as oxidation and apoptosis, thereby reducing tissue damage (Figure 5) (36). A recent study discovered that the phycocyanin peptide segment obtained by the complex enzyme action of phycocyanin could inhibit oxidative stress and inflammatory response induced by TGF-β1 to protect the lung (37).

Phycocyanin exhibits potential application value in multiple fields. In recent years, researchers have conducted a series of *in vitro*, mouse, and clinical experiments (Table 3). However, the formulation and dosage of its compatibility therapy still need to be improved based on clinical research. As a new food resource, fresh *Spirulina* can furthest preserve the biological activity of phycocyanin. Consuming it based on the recommended amount of the dietary nutrition tower and food standards is expected to exert a preventive effect to a certain degree.

**Table 3:** Biological effects of phycocyanin on diseases.

**Disease**	**Biological effect**	**Reference**
Inflammatory bowel disease	Phycocyanin affects the TLR4/NF-κB pathway through intestinal microflora and has a protective effect on intestinal damage in mice caused by high-dose radiation.	(55)
Atherosclerosis	Phycocyanin promotes the expression of CD59 in mice and delays or inhibits the development of atherosclerosis.	(56, 57)
Senile dementia	Phycocyanin αβ-dimer interacts with β-secretase and inhibits the formation of amyloid precursor proteins.	(58)
Ischemic brain injury	Phycocyanin can restore the expression levels of MBP and CNPase in ischemic rats and promote the expression of HO-1.	(59)
Non-alcoholic fatty liver disease	Phycocyanin inhibits the NF- κB pathway, lowers the proportion of antigens on the lymphocyte surface (CD4+/CD8+), upregulates the expressions of AMPK phosphorylation and the transcription factor peroxisome proliferator-activated receptor (PPAR) α, and downregulates the expression of sterol-regulatory element binding protein (SREBP) -1C.	(60, 61)
Alcoholic fatty liver disease	Phycocyanin promotes the proliferation of serum T cells, restrains levels of serum ALT, AST, and MDA, and augments the expression of SOD in the liver.	(62)
Pulmonary fibrosis	Phycocyanin abates the number of bacteria associated with inflammation and significantly increases the number of bacteria and probiotics produced by short-chain fatty acids (SCFAs). Moreover, PC can diminish the expression of inflammatory factors induced by bleomycin and suppress pulmonary fibrosis.	(63, 64)

### 3.3 Composition and nutrition values of *Spirulina* cell wall

The cell wall of *Spirulina* belongs to a type of plant cell wall, and it is a kind of crude fiber. Simultaneously, it has the characteristics of the gram-negative bacterial cell wall. It is mostly composed of peptidoglycan and contains pectin, mucopolysaccharides, cell wall acid, diaminohexanoic acid, and cellulose (38, 39). Polysaccharides in the cell wall of *Spirulina* (such as peptidoglycans and hemicellulose) can be considered prebiotics. After resisting digestion by stomach acid and trypsin, they reach the large intestine and provide nutrients for the growth of probiotics. These probiotics maintain a balance of intestinal microflora, enhance the immune system, mitigate intestinal inflammation, and promote food digestion (40, 41). Moreover, cell wall polysaccharides possess the characteristics of dietary fiber, which can scale up fecal volume, improve bowel movements, and alleviate constipation problems. Additionally, dietary fiber is conducive to controlling blood sugar and cholesterol levels. The cell wall of *Spirulina* is composed of some trace elements, such as calcium, magnesium, and zinc. These elements are released by the cell wall in the intestine and absorbed by the human body (42).

### 3.4 Glucosylglycerol and its health effects

Discovered in Japanese traditional fermented food (sake), glucosylglycerol (GG) is a type of glycoside compound formed by the reaction and linking between glycerol molecules and glucose molecules through glycosidase. GG has been reported to have various health benefits, such as treating allergic respiratory diseases, protecting conjunctiva, lowering blood sugar, and curbing visceral fat accumulation (43, 44). Luo et al. cultivated *Spirulina* under high salt stress, obtained *Spirulina* containing GG, and recovered part of phycocyanin after extracting GG, making full use of raw materials (43, 45). Fresh *Spirulina* cells can be cultured under high osmotic pressure and stored after concentration. Mixing *Spirulina* with water at a specific ratio and allowing it to settle for a while can result in an extracted liquid rich in GG for consumption. This process expands the application prospects of fresh *Spirulina* (Table 4).

**Table 4:** Preparation of fresh *Spirulina* rich in GG.

Stage	Cultivation parameter
1. Vaccination	Configure Zarrouk medium kits and inoculate the seed solution of *Spirulina* into a photobioreactor with an inoculation density of 0.15 g/L.
2. Illumination cultivation	Constant temperature: 28°C, continuous illumination, light intensity: 200 μ Mol/(m2 • s), ventilation rate: 0.2vvm, the concentration of CO_2_ in the mixed gas is a volume ratio of 2%, and continuous cultivation for 3–5 days.
3. GG generation stage	The light-to-dark ratio is 12 h: 12 h, with a temperature of 32°C under light and 23°C under dark; the ventilation rate is 0.2 vvm; the CO_2_ concentration is 5% by volume in the mixed gas; sodium chloride is added to the culture medium, with a final concentration of 150 mmol/L; the incubation continues for 3–5 days.
4. GG growing stage	The cultivation conditions are almost the same as in stage 3, except that sodium chloride is added additionally with a final concentration of 500 mmol/L, and the cultivation lasts for 3 days.
5. Cell harvesting	Cells are concentrated and stored after filtration.
6. Dilution with clean water	After diluting with water several times, the GG content is measured to reach more than 10% of the dry weight of cells.

## 4 Application of fresh *Spirulina* in food processing

Fresh *Spirulina* is extensively applied in various types of food processing. It lends nutritional value and special taste to food. Adding fresh *Spirulina* to noodles and bread can elevate their protein content, increase nutritional sources for vegetarians, and give cooked wheaten food a distinctive color and taste (46). Fresh *Spirulina* can also be added to cookies and cakes, endowing these desserts with a natural green color. For various sports, hypoglycemic, and flavored drinks, fresh *Spirulina* can incorporate nutritional value into these functional products and provide additional vitamins and minerals (42). *Spirulina* tea is a combination of *Spirulina* and tea and has both the unique nutritional value of *Spirulina* and the fresh taste of tea, making it a healthy and delicious beverage (47). Moreover, fresh *Spirulina* can be added to ice creams, fruit juices, and seasonings, rendering them special colors and flavors to meet consumers’ needs.

### 4.1 *Spirulina* pasta

*Spirulina* has a high content of proteins, vitamins, and minerals. The addition of *Spirulina* can significantly improve the nutritional value of pasta, which is especially essential for vegetarians and those who need to increase their protein intake. Adding *Spirulina* can alter the structure and characteristics of food (48). For example, increasing proteins and polysaccharides can advance the elasticity and taste of pasta, making it softer and more elastic. Furthermore, *Spirulina* can augment the viscosity of noodles and improve the extensibility of dough, contributing to the operability and processing performance during pasta making.

In the manufacturing process of microalgae noodles, *Spirulina* is usually mixed with flour to make the dough, which is then compressed, cut, or rolled to form the shape of the noodles. To ensure the texture, taste, and nutritional balance of the noodles, the addition (dry basis) is normally between 0.1 and 1.0% of the flour (49, 50).

Bread is a most common cereal staple in Europe and America. Adding fresh *Spirulina* to bread can enhance the content of vitamins, minerals, and other nutrients. Moreover, algae play a water retention role in bread, which can postpone the starch aging of bread and extend its shelf life. In the production of microalgae bread, the fermentation process and baking temperature are crucial to retain the nutritional components of *Spirulina* without altering the taste and flavor of the bread. Table 5 and show the raw materials, formula, and end product of a kind of microalgae bread developed by the Gansu Microalgae Technology Innovation Center.

**Table 5:** A recipe for *Spirulina* bread.

Material	Content (g/100 g)	Material	Content (g/100 g)
High-quality flour	74.5	Skimmed milk powder	2
Sugar and starch sugar	5	Yeast nutrient solution	0.5
Egg	10	Bread improvement additives	1
Vegetable oil	4	Yeast	2
Algal mud (dry basis)	1	MSG and salt	Appropriate amount

### 4.2 *Spirulina* beverage

Fresh *Spirulina* is widely adopted in microalgae nutritional and healthy beverages, especially in sports, hypoglycemic, and flavored drinks. However, fresh *Spirulina* has a grassy smell, which may be unacceptable for some consumers (51, 52). Some measures can be adopted to mask or mitigate this smell to improve the taste and acceptance of products, for example, using natural spices, such as vanilla, lemon, orange peel, and mint. They can augment the complexity of flavored beverages, making them tastier. Meanwhile, *Spirulina* can be combined with flavor components, such as fruit juices, plant berries, and nut sauces, to cover the grassy smell and heighten flavor diversity. In addition, the addition of *Spirulina* should be adjusted to moderate the taste of the product and assuage the perception of the grassy smell.

### 4.3 Other food applications

*Spirulina* dairy products are crafted by incorporating fresh, deodorized *Spirulina* into the dairy manufacturing process at a precise ratio. To illustrate, fresh *Spirulina* is enriched with yeast and a carbon source (glucose), undergoing fermentation to eliminate any odor, followed by pH adjustment. This process yields a blue treatment liquid abundant in phycocyanin. In formulating regular milk, 25% of this vibrant blue liquid is seamlessly integrated. With the addition of protein stabilizers and subsequent pasteurization, the *Spirulina* milk is born. A probiotic *Spirulina* yogurt, rich in carotenoids, is prepared using fresh *Spirulina*, devoid of any food additives such as stabilizers or acidity regulators (53, 54). Remarkably, the fresh *Spirulina* exhibited accelerated growth of *L. acidophilus* during fermentation and sustained its viability even during storage. These methods serve as a versatile foundation for the formulation of a multitude of innovative products.

## 5 Conclusion

Fresh *Spirulina* is a novel product form distinct from dried *Spirulina* powder, offering notable advantages in terms of nutritional composition and content, as well as functional effects. Compared to natural cow’s milk, fresh *Spirulina* contains a more comprehensive amino acid profile, elevating its significance as a valuable source of protein. Additionally, its low-fat and lactose-desensitizing properties broaden the potential applications of fresh *Spirulina*. The functional components of fresh *Spirulina*, represented by phycocyanin, functional crude fiber, and glucosylglycerol, exhibit promising efficacy in improving immunity and antioxidant capacity and maintaining intestinal health, garnering considerable attention and acclaim from scientists and international organizations worldwide. Consequently, fresh *Spirulina* is increasingly incorporated into diverse food production endeavors. In order to fully leverage the nutritional advantages of fresh *Spirulina*, expanding theoretical and applied research is essential in areas such as production reactors, cold sterilization, preservation process, and flavor blending.
